# The host jasmonic acid pathway regulates the transcriptomic changes of dodder and host plant under the scenario of caterpillar feeding on dodder

**DOI:** 10.1186/s12870-019-2161-8

**Published:** 2019-12-04

**Authors:** Yan Qin, Jingxiong Zhang, Christian Hettenhausen, Hui Liu, Shalan Li, Guojing Shen, Guoyan Cao, Jianqiang Wu

**Affiliations:** 1 Department of Economic Plants and Biotechnology, Yunnan Key Laboratory for Wild Plant Resources, Kunming Institute of Botany, the Innovative Academy of Seed Design, Chinese Academy of Sciences, Kunming, 650201 China; 2 Xingyi Normal University for Nationalities, No.1 Xingyi Road, Xingyi City, 562400 Guizhou China; 3 University of Chinese Academy of Sciences, Beijing, 100049 China

**Keywords:** Caterpillar, *Cuscuta*, Defense, Dodder, Insect herbivory, Jasmonic acid, Systemic signal, Tobacco, Transcriptome

## Abstract

**Background:**

Dodder (*Cuscuta* spp., Convolvulaceae) species are obligate leaf- and rootless parasites that totally depend on hosts to survive. Dodders naturally graft themselves to host stems to form vascular fusion, from which they obtain nutrients and water. In addition, dodders and their hosts also exchange various other molecules, including proteins, mRNAs, and small RNAs. It is very likely that vascular fusion also allows inter-plant translocation of systemic signals between dodders and host plants and these systemic signals may have profound impacts on the physiology of dodder and host plants. Herbivory is a common biotic stress for plants. When a dodder parasite is attacked by lepidopteran insects, how dodder responds to caterpillar feeding and whether there are inter-plant communications between the host plants and the parasites is still poorly understood.

**Results:**

Here, wild-type (WT) tobacco and a tobacco line in which jasmonic acid (JA) biosynthesis was silenced (AOC-RNAi) were used as the hosts, and the responses of dodders and their host plants to herbivory by *Spodoptera litura* caterpillars on the dodders were investigated. It was found that after caterpillar attack, dodders grown on AOC-RNAi tobacco showed much a smaller number of differentially expressed genes, although the genotypes of the tobacco plants did not have an effect on the simulated *S. litura* feeding-induced JA accumulation in dodders. We further show that *S. litura* herbivory on dodder also led to large changes in transcriptome and defensive metabolites in the host tobacco, leading to enhanced resistance to *S. litura*, and the JA pathway of tobacco host is critical for these systemic responses.

**Conclusions:**

Our findings indicate that during caterpillar attack on dodder, the JA pathway of host plant is required for the proper transcriptomic responses of both dodder and host plants. This study highlights the importance of the host JA pathway in regulating the inter-plant systemic signaling between dodder and hosts.

## Background

Plants and insects have coevolved for hundreds of millions of years. Almost all parts of plants (leaves, roots, stems, flowers, and seeds) can be attacked by insects. Chewing insects, such as certain beetles and caterpillars, rapidly remove and ingest plant tissues. Piercing-sucking insects use their mouthparts to either lacerate cells and suck the liquid contents (as do thrips) or to directly suck the intercellular contents in the phloem tissues (as do aphids and whiteflies). Plants are equipped with sophisticated defense systems to resist insect herbivores through activating signaling networks and subsequently synthesizing defensive compounds [1, 2]. Wounding inflicted by insect feeding is recognized by plants; moreover, specific defense responses are triggered by herbivore-associated elicitors, such as fatty-acid amino acid conjugates (FACs) in the oral secretions (OS) of lepidopteran larvae, and certain components in the salivary fluids of piercing-sucking insects [3, 4]. Ca^2+^, mitogen-activated protein kinase (MAPK), and phytohormone signaling are involved in the regulation of subsequent transcriptomic reconfigurations, leading to accumulation of defensive metabolites [5, 6].

The jasmonic acid (JA) signaling pathway plays a central role in modulating plant defense responses to many, if not all, insect herbivores, including lepidopteran insect larvae [2]. Greenhouse and field studies have shown that plants impaired in JA biosynthesis or signaling have highly compromised resistance to herbivory, due to diminished defensive metabolites. For example, silencing the *COI1* gene, which encodes the receptor of the JA-Ile (JA-isoleucine, the actual signaling jasmonate), in the wild tobacco *Nicotiana attenuata* led to dramatically decreased concentrations of nicotine, caffeoylputrescine, diterpene glycosides and the activity trypsin proteinase inhibitors (TPIs), and these *COI1*-silenced plants were very susceptible to insect herbivory [7]. In Arabidopsis (*Arabidopsis thaliana*), MYC2, MYC3, and MYC4 are three basic helix-loop-helix transcription factors having additive functions in controlling various jasmonate-induced responses. The triple mutant *myc2 myc3 myc4* was found to contain almost no glucosinolates (the anti-insect compounds in crucifers) and the larvae of the generalist *Spodoptera littoralis* grew much bigger on *myc2 myc3 myc4* than on the wild-type (WT) Arabidopsis [8].

Wounding and herbivory elicit defense responses not only in the damaged leaves (local) but also in the other undamaged ones (systemic), indicating that a systemic signal is induced in the wounded or herbivore-damaged tissues and the signal can be translocated to the other parts of the whole plant to activate defense [5]. Systemic signaling was discovered first in tomato (*Solanum lycopersicum*) [9]: Wounding a tomato leaf not only elevated the activity of proteinase inhibitor I (PI I), an anti-digestive metabolite that inhibits insect midgut protease activity, in this local leaf, but also enhanced the PI I activity systemically in the undamaged leaves. Since then, much research effort has focused on the identification of the systemic signal and the mechanisms by which the signal is generated, translocated, or perceived in distal leaves. In an elegant study, Li et al. [10] used a JA biosynthesis mutant and a JA signaling mutant of tomato for reciprocal grafting with WT tomato plants, and it was found that JA or a JA-regulated metabolite is required for the long-distance signaling. In addition to the JA pathway, electrical signals, reactive oxygen species, and glutamate receptors are also involved in systemic signaling following wounding or induced by herbivory [11–13].

Approximately 1% of flowering plants are parasites [14]. Using a special organ, the haustorium, plant parasites attach to their hosts to extract water and nutrients, resulting in retarded growth and sometimes even death of the host plants. Some parasites, such as *Orobanche* spp., *Striga* spp. (both Orobanchaceae), and *Cuscuta* spp. (Convolvulaceae), are notorious parasitic weeds causing large losses in agriculture and horticulture in many parts of the world [15]. The genus *Cuscuta* contains ca. 200 species distributed worldwide [16], and are commonly named dodders. Having no roots and leaves, dodders totally depend on their host plants to obtain water and nutrients. Using an RNA-seq approach, Kim et al. [17] identified more than 9000 different mRNAs from the Arabidopsis host in *C. pentagona* and more than 8000 mRNAs from *C. pentagona* in the Arabidopsis host. Small-RNA sequencing also revealed that 76 *Cuscuta campestris*-expressed small-RNAs were enriched at the interface between the Arabidopsis stem and the dodder haustoria, and that some of them are miRNAs functioning as suppressors of host defense against dodder parasites [18]. Green fluorescence protein (GFP) expressed in tobacco (*Nicotiana tabacum*) can be detected in the parasitizing dodders [19], suggesting that proteins are also likely to traffic between dodders and their hosts.

Recently, dodders were found to transmit herbivory-induced systemic signals. When two or more hosts are simultaneously parasitized by a single dodder plant (*Cuscuta australis*), and one of the hosts is wounded or subjected to caterpillar feeding, the dodder transmits wounding- or caterpillar feeding-induced systemic signals among the hosts, activating systemic defense responses in the undamaged plants and enhancing their resistance to subsequent infestation by lepidopteran larvae [20]. Importantly, comparison between the systemic signals sent by Arabidopsis WT and the *dde2–2* mutant (deficient in JA biosynthesis) indicated that the JA pathway plays an important role in regulating the production or transmission of the systemic signals. Similarly, feeding of green peach aphids (*Myzus persicae*) on dodder *C. australis* resulted in up- or down-regulation of more than 1000 genes in the host soybean plant (*Glycine max*), and the soybean host exhibited increased resistance to both cotton leafworm (CLW, *Spodoptera litura*) and soybean aphid (*Aphis glycines*) [21].

Some species of insects feed on dodder plants, and these are mainly aphids and curculionid weevils of the genus *Smicronyx* (Coleoptera, Curculioninae) [22, 23]. Little is known about how dodder and its host plant respond to feeding by lepidopteran caterpillars. In this study, a *Cuscuta campestris*-tobacco interaction system was used to investigate whether feeding by the chewing insect CLW on the dodder parasite is able to activate responses in both plants in the parasitization system, and whether the interactions between host and dodder in their defense against caterpillar feeding are regulated by the host JA pathway. We found that although CLW feeding on dodder activated transcriptomic changes in the parasite, much stronger transcriptomic reconfigurations were detected in the tobacco host, leading to enhanced resistance to CLW in tobacco. Importantly, the JA pathway in tobacco not only played a critical role in shaping the systemic response in the tobacco plant, but also influenced the dodder transcriptome.

## Results

### Response of dodder to CLW feeding

**Fig. 1:**
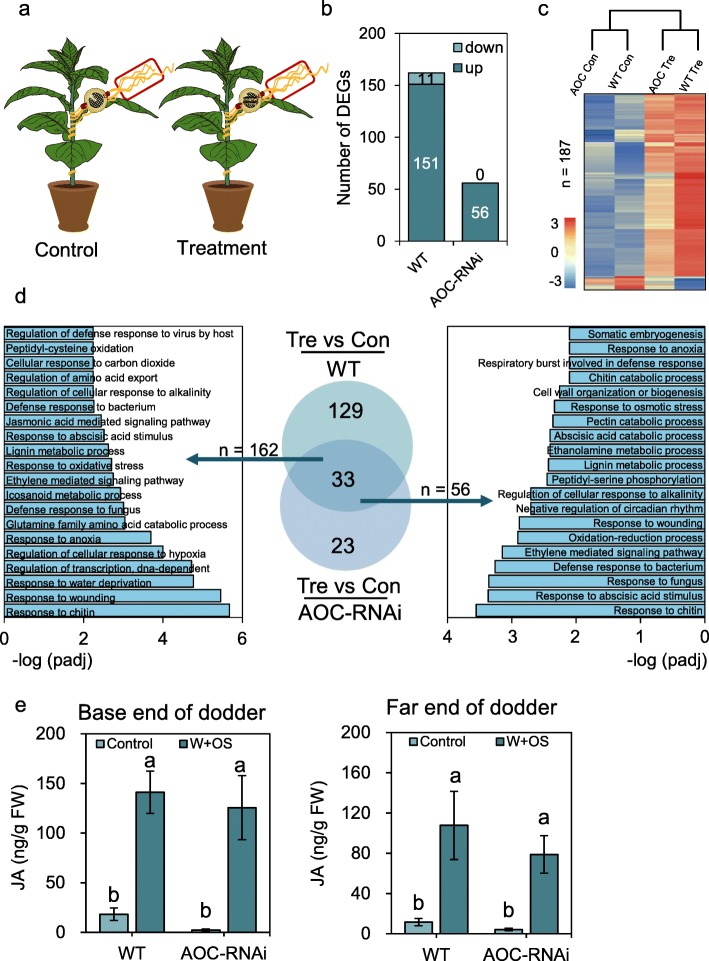
Changes in transcriptome profiles and JA levels in dodders following CLW herbivory on dodders. **a** Illustration of the experimental setup. Dodders were grown on WT and AOC-RNAi tobacco plants. Empty clip cages and clip cages each containing two CLW larvae were fixed to the dodders (one clip cage for each dodder) to form the control and treatment groups, respectively. After 12 h, dodders from both the control and treatment groups were collected for RNA-seq analysis or quantification of JA. Red boxes indicate the sampling sites. **b** The numbers of DEGs in dodders grown on WT and AOC-RNAi tobacco. **c** Heatmap and dendrogram analysis of transcriptomes of dodders (Con = control; Tre = treatment). **d** GO and Venn diagram analysis on the DEGs in dodders. **e** JA content in dodders after being treated with simulated herbivory. Dodder stems were severed with a scalpel. Leaving segments of dodder stems attached to the hosts (base end of dodder), and immediately CLW OS were pipetted to the fresh wounds; OS were also immediately applied to the wounds of the detached dodder stems (far end of dodder). The dodder stems were harvested 1 h after the treatment. Untreated dodder stem segments were also harvested at the same time to serve as controls. Data are given as means ± SE. Different letters indicate statistically significant differences (*P* < 0.05, one-way ANOVA followed by Duncan’s test, *n* ≥ 4)

*C. campestris* was used in this study, as this species grows well on tobacco. We first investigated how dodder responds at the transcriptome level to CLW feeding. In addition to the WT tobacco as the host, an AOC (*allene oxide cyclase*)-RNAi transgenic tobacco line [24], in which JA levels were very low (Additional file 1: Figure S1), was also used to determine the influence of the host JA pathway on the response of dodder to CLW feeding (illustrated in Fig. 1a). Notably, although host JA signaling seems to be involved in host defense against the dodder parasite [25], we did not observe differential growth of dodder *C. campestris* on WT and AOC-RNAi tobacco (Additional file 1: Figure S2).

After CLW had been feeding on the dodders for 12 h, samples were taken from the dodders (three biological replicates for each group). The extracted RNA samples were analyzed with RNA-seq (Additional file 2: Table S1). We found 162 differentially expressed genes (DEGs) in the dodders grown on WT tobacco following CLW herbivory and more than 90% of these DEGs (150) were up-regulated; in contrast, in dodders parasitizing AOC-RNAi tobacco, only 56 DEGs were detected (Fig. 1b; Additional file 3: Table S2), and these were all up-regulated (Fig. 1b). Dendrograms based on clustering of all the DEGs from dodders at different treatments were then generated to depict the overall comparative profiles of these transcriptomes (Fig. 1c). The transcriptome profiles of dodders growth on WT and AOC-RNAi tobacco showed relatively high similarities under both control and treatment condition. Next, we used Venn diagram analysis to compare the 162 and 56 DEGs induced in dodder, which were grew on WT and AOC-RNAi tobacco, respectively, and it was found that only 33 genes were common DEGs (Fig. 1d). Gene ontology (GO) analysis was also performed to identify the enriched biological processes from the DEGs. Among the 162 DEGs obtained from the dodders grown on WT tobacco, the terms “response to chitin”, “response to wounding”, “response to water deprivation”, “response to jasmonic acid stimulus”, and “jasmonic acid mediated signaling pathway” were enriched (Fig. 1d; Additional file 3: Table S2). The GO terms “response to chitin”, “response to abscisic acid stimulus”, “ethylene mediated signaling pathway”, “response to wounding”, and “defense response to bacterium” were enriched in the DEGs from the dodder grown on AOC-RNAi tobacco (Fig. 1d; Additional file 3: Table S2).

These results prompted us to examine whether the host JA pathway affects the dodder transcriptome even under normal conditions. Strikingly, we found 351 DEGs between the dodders growing on AOC-RNAi and WT tobacco, when dodders were untreated at all (Additional file 4: Table S3). Most of these DEGs (AOC-RNAi/WT) showed negative values (286 out of 351), indicating that the host JA pathway is required for promoting the basal transcript levels of many dodder genes. GO analysis indicated that “response to water deprivation”, “seed dormancy process”, “proton transport”, “response to cold”, and a few processes related to DNA replication were enriched (Additional file 4: Table S3).

Mechanical wounding and herbivory-induced JA plays a critical role in activating defenses in plants [1, 2]. Since insect feeding behavior is hard to control, it is difficult to use actual insect feeding to reproducibly induce short-term responses, including JA responses. Therefore, we simulated CLW herbivory on dodder by cutting the dodder stems with a scalpel and leaving 5-cm segments of dodder stems on the hosts and applying CLW OS to the fresh wounds of dodder. One hour later, the dodder segments on WT and AOC-RNAi tobacco were harvested and JA contents were quantified. We found that the levels of JA tended to be lower in dodders grown on AOC-RNAi tobacco than those grown on WT tobacco before and after simulated CLW herbivory, but no statistical differences were detected (Fig. 1e). To rule out the possibility that JA in dodder was transported from host plants, we treated the severed dodder stems (not attached to the hosts) with CLW OS, and the stems (5 cm from the detach points) were harvested 1 h after treatment. Wounding and applying OS highly increased the JA levels in dodders grown on both WT and AOC-RNAi tobacco (Fig. 1e). Thus, dodder is able to synthesize JA, although it is only stem tissue, and host JA pathway does not have a strong effect on the basal and induced JA in dodder.

All these data suggest that the host JA pathway plays a role in regulating the transcriptome of the parasitizing dodder under the normal and insect-attack scenarios.

### Response of host plant to CLW feeding on dodder

**Fig. 2:**
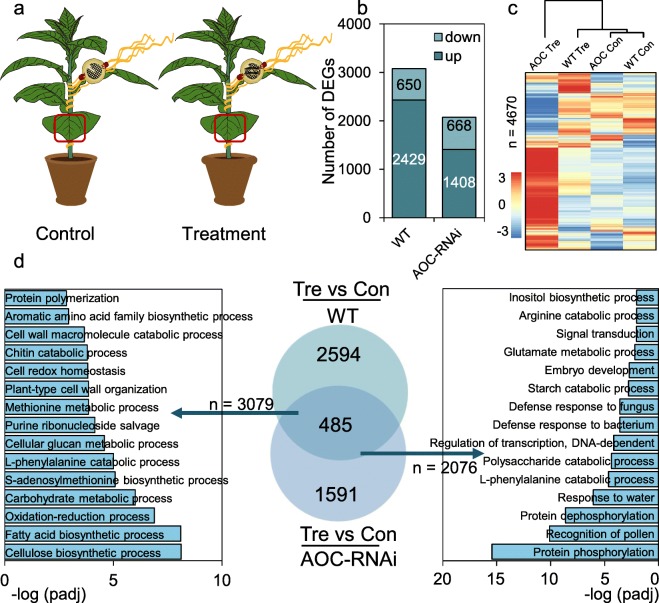
The DEGs in WT and AOC-RNAi tobacco host plants induced by CLW feeding on dodders. **a** Illustration of the experimental setup. Dodders were grown on WT and AOC-RNAi tobacco plants. Empty clip cages and clip cages each containing two CLW larvae were fixed to the dodders (one clip cage for each dodder) to form the control and treatment groups, respectively. After 12 h, leaves of WT and AOC-RNAi tobacco from the control and treatment groups were collected for RNA-seq analysis. Red box indicates the sampling sites. **b** The numbers of DEGs in WT and AOC-RNAi tobacco hosts before and after dodders were treated with CLW herbivory (Con = control; Tre = treatment). **c** Heatmap and dendrogram analysis of transcriptomes of the WT and AOC-RNAi tobacco. **d** GO and Venn diagram analysis on the DEGs from WT and AOC-RNAi tobacco(Con = control; Tre = treatment)

To determine whether the host plant tobacco, which was not attacked by insects but connected with dodder, also responds to CLW feeding on dodder, we examined the transcriptomic changes in the tobacco host (Fig. 2a; Additional file 5: Table S4). In WT and AOC-RNAi tobacco, 12 h of CLW feeding on dodder up-regulated 2429 and 1408 genes, respectively (Fig. 2b; Additional file 6: Table S5). Many less genes (650 and 668, respectively) were downregulated in the WT and AOC-RNAi host (Fig. 2b; Additional file 6: Table S5). Dendrogram analysis indicated that under normal conditions (control) WT and AOC-RNAi plants had relatively similar transcriptomes, and after herbivory treated on their respective parasitizing dodders, while WT transcriptome still grouped with those of control WT and AOC-RNAi plants, AOC-RNAi transcriptome was remote from the others (Fig. 2c). Consistent with the large differences between the transcriptomes of WT and AOC-RNAi plants after herbivory on dodders (Fig. 2c), Venn diagram analysis indicated that only 485 genes were commonly regulated between the DEGs identified from the herbivory-treated WT and AOC-RNAi host plants (Fig. 2d). GO analysis indicated that “cellulose biosynthetic process”, “fatty acid biosynthetic process”, “oxidation-reduction process”, and “*S*-adenosylmethionine biosynthetic process” were highly enriched GO terms in the WT tobacco samples, while “protein phosphorylation”, “protein dephosphorylation”, “recognition of pollen”, and “response to water” were among the highly enriched GO terms in the AOC-RNAi samples (Fig. 2d; Additional file 6: Table S5). Thus, CLW caterpillar feeding on dodder induces large transcriptomic reconfiguration in the host plant, and the host JA pathway is required for the proper host transcriptomic changes.

**Fig. 3:**
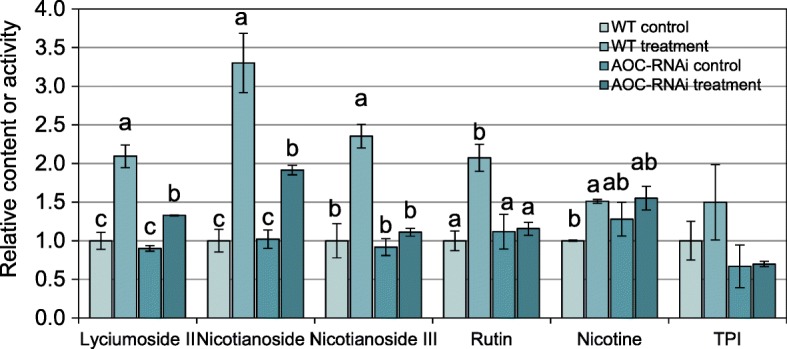
Secondary metabolites in WT and AOC-RNAi tobacco host plants following 48 h of CLW feeding on their dodder parasites. The experimental setup is shown in Fig. 1a. Dodders were grown on WT and AOC-RNAi tobacco plants. Empty clip cages and clip cages each containing two CLW larvae were fixed to the dodders (one clip cage for each dodder) to form the control and treatment groups, respectively. After 48 h, WT and AOC-RNAi tobacco leaves from the control and treatment groups were collected for analysis of secondary metabolites. Different lowercase letters represent statistical significance (*n* = 5; one-way ANOVA with Duncan’s test). Error bars are standard errors

Next, we sought to determine whether CLW feeding on dodder leads to the accumulation of host defensive metabolites. After 60 h of CLW feeding on dodder, the concentrations of lyciumoside ІІ, nicotianoside І, and nicotianoside Ш (three anti-insect 17-hydroxygeranyllinalool diterpene glycosides [26]) in the WT tobacco plant hosts had increased at least 1-fold. However, in AOC-RNAi plants these metabolites were not induced or induced to a lesser extent, compared with those in WT tobacco (Fig. 3). Similar results were obtained for rutin, and TPI activity tended to be induced in WT but not at all in the AOC-RNAi tobacco (Fig. 3). Nicotine content increased about 51% in the WT tobacco, while no significant changes were detected in AOC-RNAi tobacco following CLW feeding on the parasitizing dodder (Fig. 3).

**Fig. 4:**
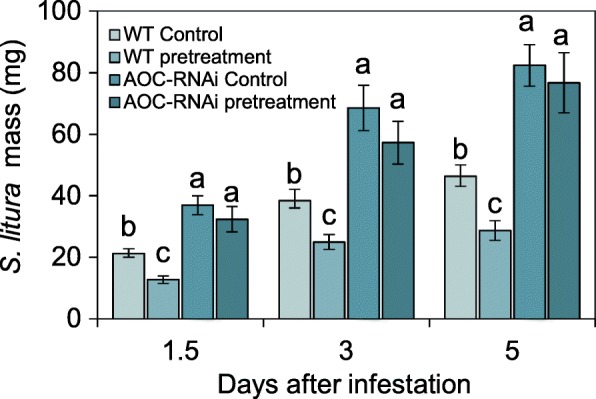
Resistance of tobacco host plants to CLW larvae after dodders were pretreated with CLW herbivory. The experimental setup is shown in Fig. 1a. Dodders were grown on WT and AOC-RNAi tobacco plants. Dodders parasitizing WT tobacco were subjected either to herbivory by two CLWs (pretreatment) or kept untreated (control) for 48 h. After 48 h, a new CLW larva was introduced to each tobacco and the masses of these larvae were recorded on day 1.5, 3, and 5. Different lowercase letters represent statistical significance (*n* = 15; one-way ANOVA with Duncan’s test). Error bars are standard errors

To investigate whether CLW feeding on dodder increases levels of host defense, a CLW growth assay was conducted on the WT and AOC-RNAi tobacco plants infested with dodders, where these parasites had been either pretreated with CLW feeding for 48 h (pretreatment group) or left untreated as controls. We found that compared with the CLWs feeding on tobacco of the control group, CLWs feeding on the tobacco in the group in which dodder had been pretreated were 40, 35, and 31% smaller on days 1.5, 3, and 5, respectively (Fig. 4). In contrast, the masses of the CLWs feeding on AOC-RNAi tobacco were not altered by the herbivory pretreatment of the parasitic dodders, and the insects grew much larger on AOC-RNAi plants than on the WT plants (Fig. 4). We concluded that caterpillars feeding on the dodder induce certain systemic signals in the plants, which travel to host plants and elevate host resistance to subsequent caterpillar attack, and that the JA pathway of the host plant is required for activating the defenses.

### Dodder response to simulated CLW feeding on tobacco host

The strong response of the tobacco host to CLW feeding on dodder led us to investigate whether CLW feeding on the tobacco host also changes the dodder transcriptome. The defense mechanisms against insects in AOC-RNAi tobacco plants are highly compromised, and CLWs consume substantially more leaves on these plants [24] than on the WT tobacco. Therefore, in order to standardize the treatment, in this experiment we did not use real CLW feeding to treat these plants. Instead, tobacco plants were treated with continuous simulated herbivory: WT and AOC-RNAi tobacco hosts were wounded with a pattern wheel to create a row of wounds and CLW OS were immediately applied to the wounds, and this was repeated every 2 h for another five times. Dodder samples were collected 2 h after the last simulated herbivory treatment and used for RNA-seq analysis (Additional file 7: Table S6). We found only 12 and 7 DEGs in the dodder parasites growing on the WT and AOC-RNAi tobacco, respectively (Additional file 8: Table S7). The numbers of these DEGs are too small to be genuine; they are most likely artifacts of random noise from RNA-seq analysis (SEQC MAQC-III Consortium, 2014). Thus, dodder did not respond, or responded only very little, to simulated CLW feeding on host plants at the transcriptome level.

To investigate whether simulated herbivory treatment on host tobacco and the JA pathway of host affect the resistance of dodder to subsequent insect attack, WT and AOC-RNAi tobacco plants were similarly pretreated with simulated herbivory (pretreatment group) or were left untreated (control group), and 48 h later CLWs were introduced to dodder plants parasitizing these tobacco plants. Consistent with the transcriptomic data, we did not detect differences in the mass of CLWs from the pretreatment and control groups (Additional file 1: Figure S3).

## Discussion

Dodders are morphologically unusual parasitic plants, which have no roots or leaves. The parasitization process of dodder can be considered to be a natural grafting, allowing the xylem and phloem of dodder respectively fuse with the xylem and phloem of host plant. How dodder adapts to environmental stresses and how dodder and its host plant interact during adaption to stresses through exchange of various molecules through vasculature, including signaling molecules, remains unclear. In this study, we show that CLW feeding on dodder not only activated small-scale transcriptomic changes and elevated JA levels in the dodder itself, but also induced large numbers of DEGs and defensive metabolites in the host plants. Furthermore, our analyses reveal that the host JA pathway has certain impact on the transcriptional regulation of dodder to herbivory, demonstrating the JA signaling plays an important role in the inter-plant communication during the response of dodder to herbivory.

We found that direct feeding of CLW on dodder changed the transcript levels of 162 genes in dodder (Fig. 1b); in contrast, more than 3000 were found to be up- or downregulated in the host tobacco. This is consistent with the results of an experiment in which in aphid feeding on dodder grown on a soybean host resulted in about 170 and 1000 genes being differentially regulated in dodder and soybean, respectively [21]. It is very likely that dodder can distinguish herbivory from both types of insect herbivory (chewing, piercing-sucking) and in turn produces the corresponding mobile signals. These signals travel to other parts of the dodder and also travel across the haustorium-host stem junctions to reach different parts of the host plant, where they activate strong transcriptomic, proteomic, and metabolic reconfigurations in the host plant, increasing its defense against insects. Our data also indicate that the host JA pathway is required for the dodder-derived systemic signals to activate defenses in the host plant, since herbivory on the dodder parasite did not elevate the resistance of the AOC-RNAi host (Fig. 4).

After CLW herbivory, dodders on the AOC-RNAi plants showed many fewer DEGs than did those on WT hosts (Fig. 1b,d), and feeding treatments also induced distinct transcriptomic changes in the AOC-RNAi and WT host plants (Fig. 2). Therefore, although the underlying mechanism is unknown, the host plant’s JA pathway influences the response of the dodder parasite to insect feeding, and the JA pathway of the host plant likely also has an effect on the dodder-derived systemic signal (Fig. 4). We concluded that the host JA pathway has a function in the communication between dodder and host during herbivore feeding on dodder. It is possible that certain previously unknown JA-dependent mobile signals from the host plant can be translocated to the dodder parasite and thus modify the defense physiology in dodder. Supporting this scenario, grafting experiments using WT and *spr-2* (a JA biosynthesis mutant) tomato plants demonstrated that the JA pathway is critical for the generation of wounding-induced mobile signals [10]. Furthermore, using the wild tobacco *Nicotiana attenuata*, Bozorov et al. [27] grafted the irAOC plants (silenced in the JA biosynthetic gene *allene oxide cyclase*) as the stocks to WT scions; it was shown that treating the irAOC stocks with simulated insect feeding elicited less nicotine, diterpene glycosides, and trypsin proteinase inhibitors in the WT scions than in the WT scions of the WT/WT control grafts.

Systemic signals induced by feeding of caterpillars or aphids on host plants activated little alteration in the dodder transcriptome [[20, 21] and this study], and consistently, simulated CLW feeding on tobacco did not change the resistance of dodder against subsequent CLW attack. The possibility that host-produced systemic signals are very weak could be ruled out, since systemic signals can pass across dodder bridges and trigger strong defense in the neighboring host [20]. Recently, the genome sequence of *C. australis* revealed losses of a large number of genes, including many important for defense against insects and pathogens [28]. Although the analysis of the *C. campestris* genome did not focus on defense-related genes [29], given the close phylogenetic relationship between *C. australis* and *C. campestris* [30], it is likely that the *C. campestris* genome has similarly experienced loss of a large number of defense-related genes. It is plausible that dodders do not respond as much as normal autotrophic plants to systemic signaling, due to loss of these defense-related genes.

Grafting between WT and *spr-2* mutant, which lacks of JA, indicated that JA or a JA-regulated metabolite is the mobile signal [10]. Similarly, in previous experiments we connected WT and *dde2–2* (JA biosynthesis mutant) Arabidopsis with tobacco through dodder bridges, and found that the JA pathway is essential for regulating the production and/or transmission of the herbivory-induced systemic signal. However, JA is very unlikely to be the mobile signal per se: The speed that the mobile signals travel far exceeds that of JA accumulation [11, 31], and furthermore, the signals can induce activation of MAPKs and accumulation of JA/JA-Ile in systemic leaves, suggesting that the signals are upstream of both the MAPK pathway and JA biosynthesis [11, 31, 32]. In this study, we found that CLW feeding on dodder resulted in large differences between the transcriptomes of WT and AOC-RNAi tobacco (Fig. 2), indicating that the JA pathway is located downstream of the caterpillar feeding-induced systemic signal and is very important for the proper response to the systemic signals. Thus, it seems that the JA pathway plays a dual role in systemic signaling. Presumably, the basal JA signaling activity (under normal conditions) is required for maintaining the level/accumulation of the systemic signal and upon wounding/caterpillar feeding the signals are somehow rapidly released from the local tissues and are translocated to other parts of the plant, where they activate both JA-dependent and -independent responses.

## Conclusions

Previously, it has been found that aphid feeding on dodder also enhanced the defense of the host plant [21]. Furthermore, when different host plants are simultaneously connected by the same dodder, caterpillar feeding-induced systemic signals from one host also activated defenses in the other hosts [20]. In this study, we show that dodder subjected to caterpillar feeding sends certain systemic signals to the host plants, and the signals are able to induce transcriptome regulation and defense in the host, enabling the host to be better protected from subsequent insect feeding. Importantly, in this process, host JA pathway plays an essential role in regulating the systemic signals which mediate the communication between host and dodder. Although dodder parasitization has a strong negative effect on host plant growth and development, our data further highlight that dodder parasitization provides the host with certain ecological benefit, even though probably subtle.

## Methods

### Plant materials and rearing of insects

The experimental research on all plants complied with institutional and national guidelines. All plants were grown in a glasshouse at the Kunming Institute of Botany, Chinese Academy of Sciences. The day/night length was ~ 16 h/8 h and the temperature was maintained at ~ 25 °C (day) and 18 °C (night). Dodder (*Cuscuta campestris*) seeds were initially purchased from an herbal medicine store in Kunming (Yunnan, China) and the species identification was kindly done by Prof. Fenggen Guo (Yunnan Agricultural University). The voucher specimens of *C. campestris* can be accessed at the Herbarium of the Kunming Institute of Botany, Chinese Academy of Sciences (accession No. 1347916). No permission was needed for obtaining and using the dodder seeds.

Seeds of tobacco (*Nicotiana tabacum* cv. Samsun, a gift from Dr. Ian T. Baldwin, Max Planck Institute for Chemical Ecology, Germany) wild-type (WT) and a homemade transgenic line named AOC-RNAi, in which both tobacco *allene oxide cyclase* (*AOC*) genes are silenced by RNA interference (RNAi) (Lu et al., 2018), were used as the hosts for dodder. The tobacco genome comprises two *AOC* genes, while eight *AOC* genes exist in *C. campestris* genome (accession numbers see Additional file 9: Table S8). Quantitative real time-PCR (qRT-PCR) analysis indicated that the RNAi construct for tobacco *AOC* silencing did not induce inter-plant RNAi silencing of the dodder *AOC* genes, probably due to the poor homology of the *AOC* genes between tobacco and dodder (Additional file 1: Figure S4). WT and AOC-RNAi tobacco seeds were germinated on agar plates containing Gamborg’s B-5 basal salt mixture (Sigma), and the seedlings were transferred to 1-L plastic pots containing 0.9 L of soil (Pindstrup Blond Gold, http://www.pindstrup.com). Germination of dodder seeds (*Cuscuta campestris*) followed Li et al. [33], and five-day-old dodder seedlings were then used to infest soybean plants (*Glycine max* cv. Huachun 6, purchased from a local seed store) to form dodder stocks. Freshly excised young vines from vigorously growing dodder stocks, about 10 cm in length, were used to infest the stems of four-week-old tobacco plants (one dodder for one host plant) and were further allowed to grow for an additional four weeks.

Eggs of cotton leafworm (*Spodoptera litura*, CLW) were purchased from the Beijing Genralpest Company (https://shop101732681.taobao.com) and the eggs were kept in a growth chamber (16 h of light at 26 °C and 8 h of dark at 22 °C) until the larvae hatched. CLW neonates were reared on Arabidopsis for 5 days before they were used for further experiments (CLW neonates show a high mortality rate on tobacco, if they are not first reared on Arabidopsis for 5 days).

### CLW herbivory and simulated CLW herbivory treatment on dodder

To assess the effect of CLW herbivory on dodder, dodder-WT tobacco and dodder-AOC-RNAi tobacco systems were cultivated as indicated above. For each dodder-host interaction system, a clip cage containing two CLWs was attached to the dodder plant. Each clip cage enclosed 10 dodder stems, and was attached about 3 cm away from the tobacco stem; as a control, empty clip cages were similarly attached to the dodders but with no insect herbivory (illustrated in Fig. 1a).

To simulate CLW herbivory on dodder, five stems from each host plant were severed with a scalpel, leaving 5-cm segments of dodder stems attached to the hosts. Immediately, CLW OS (5 μL) were pipetted to each of the fresh wounds of the dodder stems remaining on the hosts, and similarly, 5 μL of OS were applied to the wounds of the detached dodder stems. The dodder stems remained on the hosts and the detached dodder stems (5 cm from the detaching points) were harvested 1 h after the treatment. Untreated dodder stem segments at the same positions as those treated with simulated herbivory were also harvested at the same time to serve as controls.

### Simulated CLW herbivory treatment on tobacco

Dodder-WT tobacco and dodder-AOC-RNAi tobacco systems were cultivated as indicated above. For the simulated CLW herbivory treatment on tobacco plants, the fourth stem leaf from the bottom of each tobacco was wounded by rolling a pattern wheel three times on each side of the midvein in a direction parallel to the midvein, and each row of wounds was generated every 2 h on the previously wounded leaves; immediately following wounding, for each row, 20 μL of CLW oral secretions (OS) were gently rubbed into the fresh wounds.

To collect CLW OS, CLW larvae were reared on tobacco and OS were collected with a pipette and stored on ice. The OS were gently mixed and immediately aliquoted to small volumes before being stored at − 80 °C.

### CLW growth assay

WT and AOC-RNAi tobacco plants (30 each) parasitized by dodders were used to determine whether CLW feeding on dodder alters tobacco host resistance to subsequent CLW attack. These plants were evenly divided to two groups, the control and the pretreatment groups. Dodders in both groups had a single empty clip cage per dodder attached (enclosing about 10 dodder stems, about 3 cm away from the tobacco stem), and two CLW larvae were released into each clip cage in the pretreatment group. After 48 h of feeding, a new clip cage containing a single CLW larva was attached to each of the tobacco hosts, and the masses of the larvae were recorded on days 1.5, 3, and 5.

To determine whether CLW feeding on tobacco hosts changes dodder resistance, 60 WT and 60 AOC-RNAi tobacco plants infested with dodder were evenly divided to two groups. Tobacco plants from both groups were wounded to simulate CLW herbivory (as described above) or left untreated, and 48 h after the last simulated herbivory treatment, each dodder plant was infested with a single CLW larva enclosed in a clip cage. The larval masses were recorded after a further 48 h.

### Total RNA extraction, RNA-seq analysis, and qRT-PCR

Three biological replicates were taken from each group of samples. Total RNA was extracted using the TriZol reagent (Invitrogen). The concentrations of total RNA were determined on a NanoDrop spectrophotometer (Thermo Scientific) and the integrity of the total RNA was examined with gel electrophoresis. The tabacum leaf and dodder stem cDNA libraries were constructed using an Illumina kit following the manufacturer’s protocol and the resulting cDNA libraries were sequenced on an Illumina HiSeq2000 platform (paired-end sequencing; length 150 bp). Raw reads were filtered to remove low quality reads, which contained more than 10% unknown bases (N). The filtered reads from dodder and tobacco were aligned against *Cuscuta australis* [28] and *Nicotiana tabacum* (cv. TN90) [34] genome independently using HISAT2 [35] (v2.0.5) with parameters “-mp 3,1”, achieving the average ratio of the mapped reads 94% (dodder) and 81.3% (tobacco). Transcript abundances were calculated using StringTie [36] (v1.3.0). Differential gene expression was inferred based on the total mapping counts using the Bioconductor DEseq package [37], taking into account the batch effect between the different biological experiments. The assignment of differential transcription relied on the probability (p) value and Benjamini-Hochberg false discovery rate, and the former corresponded to a differential gene transcription test, while the latter was used to determine the threshold *p*-value. The thresholds applied were FDR (false discovery rate) ≤ 0.05 and the absolute value of log_2_(treatment/control) ≥ 1. Functional classification of expressed genes exploited the Gene Ontology (GO) [38, 39]. GO terms enriched by DEGs were analyzed with TopGo [40]. For each differentially expressed gene, the list of gene ID and GO number are provided. The classic p-value < 0.01 was considered to be significantly enriched. All the raw RNA-seq data can be found in the NCBI SRA database under the project ID PRJNA486679.

Five biological replicates from each group of samples were used for qRT-PCR analysis. Total RNA samples (0.5 μg) were reversed transcribed to cDNA using oligo (dT) and Superscript II reverse transcriptase (ThermoFisher Scientific). qRT-PCR was performed using a CFX Connect real-time system (Bio-Rad) using the iTaqTM Universal SYBR Geen Supermix Kit (Bio-Rad). For each analysis, a linear standard curve and a threshold cycle number versus log (designated transcript level) curve were constructed using a series dilution of a specific cDNA standard; the levels of the transcript in all unknown samples were determined according to the standard curve. Primer sequences are given in Additional file 9: Table S8.

### Quantification of secondary metabolites

Tobacco tissues were harvested and ground into fine power in liquid nitrogen. For each sample, around 200 mg of tissue were transferred to a fresh 1.5 mL microfuge tube, and 1 mL of extraction solution (40% methanol containing 0.1% acetic acid (v/v)) was added. After vortexing for 10 min, the mixtures were centrifuged at 4 °C and 16,000 *g* for 20 min. The supernatants (400 μL) were transferred to fresh tubes and centrifuged again at 4 °C and 16,000 *g* for 15 min. The nicotine, rutin, and diterpene glycosides content in the supernatants were analyzed using a high-performance liquid chromatography-tandem mass spectrometer (HPLC-MS/MS; Shimadzu LCMS8040 system) following a method published previously [41]. To quantify TPI (trypsin proteinase inhibitor) activity in tobacco leaves, a method described by Van Dam et al. [42] was used.

### Quantification of JA

For quantification of JA, around 100 mg of fresh dodder stems were ground in liquid nitrogen and 1 mL of ethyl acetate spiked with the internal standards (100 ng of D_6_-JA) was added to each sample. After a 10-min vortexing step, followed by centrifugation at 13,000 g for 15 min at 4 °C, the supernatants were evaporated to dryness in a vacuum concentrator (Eppendorf) at 30 °C. Samples were resuspended in 600 μL of 70% methanol (v/v) and again centrifuged at 13,000 g for 15 min at 4 °C to remove particles. The supernatants were analyzed on a HPLC-MS/MS (Shimadzu LCMS8040 system), following a method published previously [32].

## Supplementary information

**Additional file 1: Figure S1.** JA contents in WT and AOC-RNAi tobacco plants. WT and AOC-RNAi tobacco were infested with dodders. The leaves of tobacco were wounded by rolling a pattern wheel 6 times along the midrib (3 rolls on each side). No treatment was done to the control group. These tobacco leaves and stems were collected in 1 h and used for JA quantification. Asterisks indicate significant differences between control and treatment groups determined by Student’s *t*-test (*n* = 5; **, *p* < 0.01; ***, *p* < 0.001). Error bars are standard errors. **Figure S2**: The lengths and fresh and dry masses of dodders growth on WT and AOC-RNAi tobacco plants. Dodders were used to infest WT and AOC-RNAi tobacco plants. Four weeks after infestation, the dodders were harvested and the lengths of dodder stems (A) and their fresh and dry masses (B and C) were measured (*n* = 12; Student’s *t*-test). Error bars are standard errors. No statistical differences were found. **Figure S3**: The mass differences of CLWs on dodders of the pretreatment and control group. Dodders were growing on 30 WT and 30 AOC-RNAi tobacco plants. For the pretreatment group, 15 WT and AOC-RNAi tobacco plants were wounded with a pattern wheel to generate six rows of wounds, each row of wounds was made 2 h apart, and after each wounding treatment, CLW OS were immediately applied to the wounds. Fifteen untreated WT and AOC-RNAi tobacco plants served as controls. Forty-eight h after the last treatment, clip cages each containing one CLW were fixed to the dodders (one clip cage for each dodder), and the insect masses were recorded after another 48 h. No statistical differences were found between any groups (*n* = 15; one-way ANOVA with Duncan’s test). Error bars are standard errors. **Figure S4**: Relative *AOC* expression levels in dodder. Dodders were grown on WT and AOC-RNAi tobacco plants. The stems were harvested and the relative expression levels of dodder *AOC* genes were determined with qRT-PCR analysis. No statistical significance was found (n = 5; one-way ANOVA with Duncan’s test). Error bars are standard errors.

**Additional file 2: Table S1.** Transcriptomic changes of dodders on WT or AOC-RNAi tobacco after dodders were treated with CLW herbivory.

**Additional file 3: Table S2.** The DEGs and their GOs of the CLW-treated and control dodders growing on WT and AOC-RNAi tobacco plants.

**Additional file 4: Table S3.** The DEGs and their GOs between dodders grown on WT and AOC-RNAi tobacco plants under normal conditions.

**Additional file 5: Table S4.** WT and AOC-RNAi tobacco transcriptomic changes after dodders were treated with CLW feeding.

**Additional file 6: Table S5.** The DEGs and their GOs of WT and AOC-RNAi tobacco plants after CLW feeding on dodders.

**Additional file 7: Table S6.** Transcriptomic changes of dodders on WT or AOC-RNAi tobacco after tobacco were treated with simulated CLW herbivory.

**Additional file 8: Table S7.** DEGs of dodders after WT or AOC-RNAi tobacco plants were treated with simulated CLW herbivory.

**Additional file 9: Table S8.** Primers used for qRT-PCR.

## Data Availability

The data sets supporting the results of this article are included within the article and its additional files. RNA-seq data are available at the NCBI SRA database under the project ID PRJNA486679.

## Abbreviations

AOC: Allene oxide cyclase
DEGs: Differentially expressed genes
FACs: Fatty-acid amino acid conjugates
FDR: False discovery rate
GFP: Green fluorescence protein
JA: Jasmonate acid
MAPK: Mitogen-activated protein kinase
OS: Oral secretions
PI I: Proteinase inhibitor I
qRT-PCR: Quantitative real time-PCR
RNAi: RNA interference
TPI: Trypsin proteinase inhibitor
WT: Wild type
