# Supplementary figures and images for: The host jasmonic acid pathway regulates the transcriptomic changes of dodder and host plant under the scenario of caterpillar feeding on dodder
Source: BMC Plant Biol. 2019 Dec 4;19:540. doi: 10.1186/s12870-019-2161-8 (PMC6894313; doi:10.1186/s12870-019-2161-8)

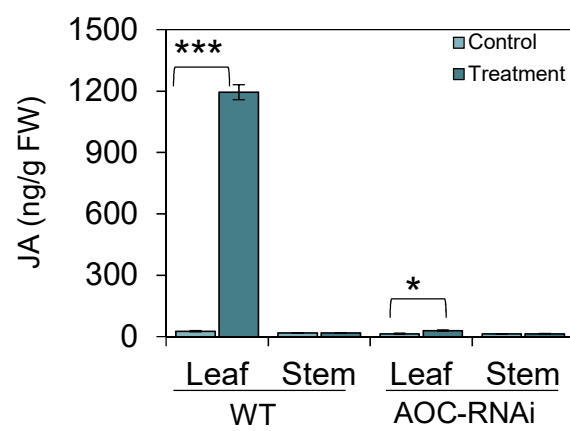

**Fig. S1**

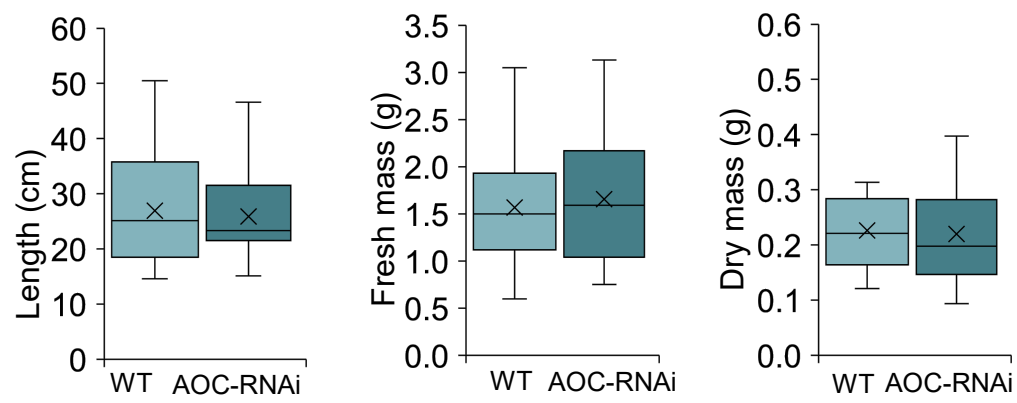

**Fig. S2**

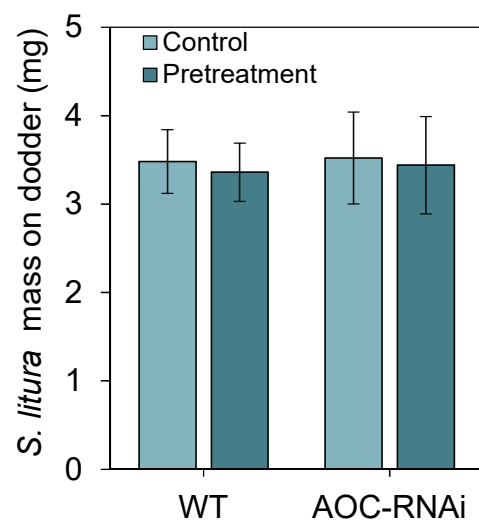

**Fig. S3**

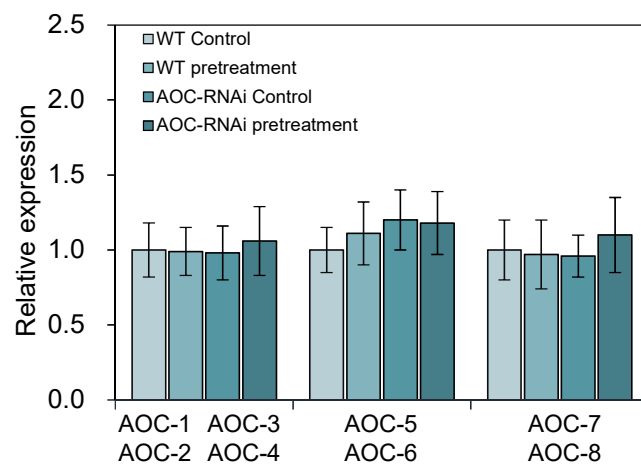

**Fig. S4**

Supplement: Supplementary file 1 — Additional file 1: Figure S1. JA contents in WT and AOC-RNAi tobacco plants. WT and AOC-RNAi tobacco were infested with dodders. The leaves of tobacco were wounded by rolling a pattern wheel 6 times along the midrib (3 rolls on each side). No treatment was done to the control group. These tobacco leaves and stems were collected in 1 h and used for JA quantification. Asterisks indicate significant differences between control and treatment groups determined by Student’s t-test (n = 5; **, p < 0.01; ***, p < 0.001). Error bars are standard errors. Figure S2: The lengths and fresh and dry masses of dodders growth on WT and AOC-RNAi tobacco plants. Dodders were used to infest WT and AOC-RNAi tobacco plants. Four weeks after infestation, the dodders were harvested and the lengths of dodder stems (A) and their fresh and dry masses (B and C) were measured (n = 12; Student’s t-test). Error bars are standard errors. No statistical differences were found. Figure S3: The mass differences of CLWs on dodders of the pretreatment and control group. Dodders were growing on 30 WT and 30 AOC-RNAi tobacco plants. For the pretreatment group, 15 WT and AOC-RNAi tobacco plants were wounded with a pattern wheel to generate six rows of wounds, each row of wounds was made 2 h apart, and after each wounding treatment, CLW OS were immediately applied to the wounds. Fifteen untreated WT and AOC-RNAi tobacco plants served as controls. Forty-eight h after the last treatment, clip cages each containing one CLW were fixed to the dodders (one clip cage for each dodder), and the insect masses were recorded after another 48 h. No statistical differences were found between any groups (n = 15; one-way ANOVA with Duncan’s test). Error bars are standard errors. Figure S4: Relative AOC expression levels in dodder. Dodders were grown on WT and AOC-RNAi tobacco plants. The stems were harvested and the relative expression levels of dodder AOC genes were determined with qRT-PCR analysis. No statistical signifi [file 12870_2019_2161_MOESM1_ESM.pdf]
